# Iodine in Diphtheria

**Published:** 1900-12-08

**Authors:** 


### IODINE IN DIPHTHERIA.

MR. HUGH TAYLOR, of Wroxham,[1] speaking from an experience of a quarter of a century, advises the use of iodine in the treatment of diphtheria. On being called to a case he generally gives a good dose of calomel, paints the throat, tonsils, back of pharynx and uvula with tincture of iodine by means of a camel's-hair brush or a swab of cotton wool, and advises the repeated inhalation of the tincture of iodine in hot water—about a teaspoonful of the tincture in a quarter of a pint of hot water—for about five minutes at a time every half-hour or so, or even in severe cases more frequently. The inhalation must be well done and the vapour must be passed through the nose and throat. Sometimes small doses of iodine may be given internally, but the medicines recommended for internal use are quinine and tincture of perchloride of iron.

[1] British Medical Journal, November 10.

